# 2-(4-Chloro­phen­yl)-6-methyl-4-(3-methyl­phen­yl)quinoline

**DOI:** 10.1107/S1600536812043954

**Published:** 2012-10-31

**Authors:** M Prabhuswamy, T. R. Swaroop, S. Madan Kumar, K. S. Rangappa, N. K. Lokanath

**Affiliations:** a Department of Studies in Physics, Manasagangotri, University of Mysore, Mysore 570 006, India; b Department of Studies in Chemistry, Manasagangotri, University of Mysore, Mysore 570 006, India

## Abstract

In the title compound, C_23_H_18_ClN, the dihedral angles between the quinoline unit and the chloro­benzene and methyl­benzene rings are 2.57 (9) and 56.06 (9)°, respectively. The crystal structure is stabilized by π–π inter­actions [minimum ring centroid separation = 3.733 (2) Å].

## Related literature

For quinolines, see: Michael (1997 ▶); Balasubramanian *et al.* (1996 ▶). For a related structure, see: Asiri *et al.* (2011 ▶).
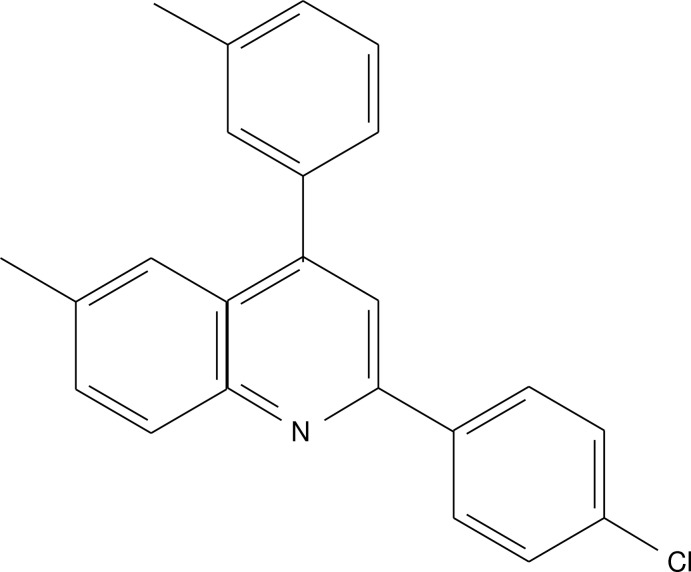


## Experimental

### 

#### Crystal data

C_23_H_18_ClN
*M* *_r_* = 343.83
Monoclinic, 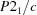

*a* = 7.982 (3) Å
*b* = 17.921 (6) Å
*c* = 12.478 (4) Å
β = 92.581 (6)°
*V* = 1783.1 (11) Å^3^
*Z* = 4
Mo *K*α radiation
μ = 0.22 mm^−1^
*T* = 293 K
0.23 × 0.22 × 0.22 mm

#### Data collection

Oxford Diffraction Xcalibur CCD diffractometer
16998 measured reflections
3392 independent reflections
2508 reflections with *I* > 2σ(*I*)
*R*_int_ = 0.044

#### Refinement

*R*[*F* ^2^ > 2σ(*F* ^2^)] = 0.057
*wR*(*F* ^2^) = 0.168
*S* = 1.04
3392 reflections
228 parameters
H-atom parameters constrained
Δρ_max_ = 0.49 e Å^−3^
Δρ_min_ = −0.45 e Å^−3^

### 

Data collection: *CrysAlis PRO* (Oxford Diffraction, 2009 ▶); cell refinement: *CrysAlis PRO*; data reduction: *CrysAlis PRO*; program(s) used to solve structure: *SHELXS97* (Sheldrick, 2008 ▶); program(s) used to refine structure: *SHELXL97* (Sheldrick, 2008 ▶); molecular graphics: *Mercury* (Macrae *et al.*, 2006 ▶); software used to prepare material for publication: *SHELXL97*.

## Supplementary Material

Crystal structure: contains datablock(s) global, I. DOI: 10.1107/S1600536812043954/zs2238sup1.cif

Structure factors: contains datablock(s) I. DOI: 10.1107/S1600536812043954/zs2238Isup2.hkl

Supplementary material file. DOI: 10.1107/S1600536812043954/zs2238Isup3.cml

Additional supplementary materials:  crystallographic information; 3D view; checkCIF report

## Figures and Tables

**Fig. 1.**
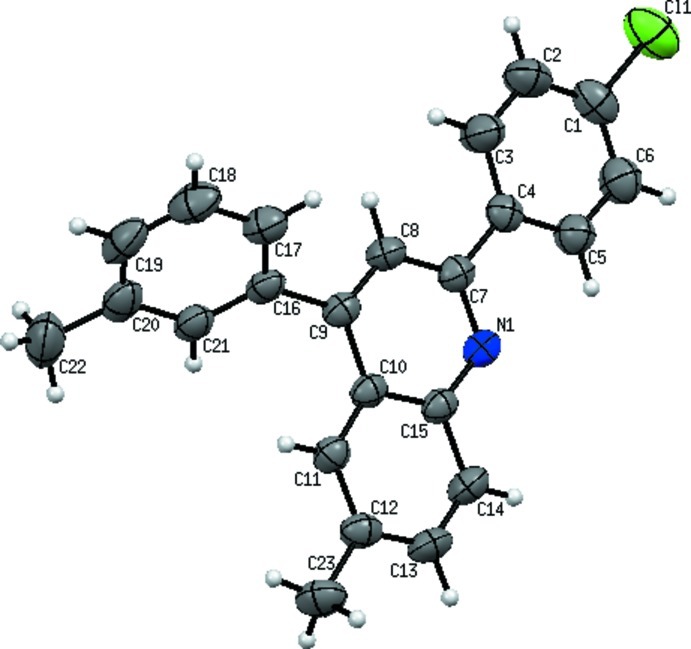
ORTEP diagram of the title compound showing 50% probability ellipsoids.

**Fig. 2.**
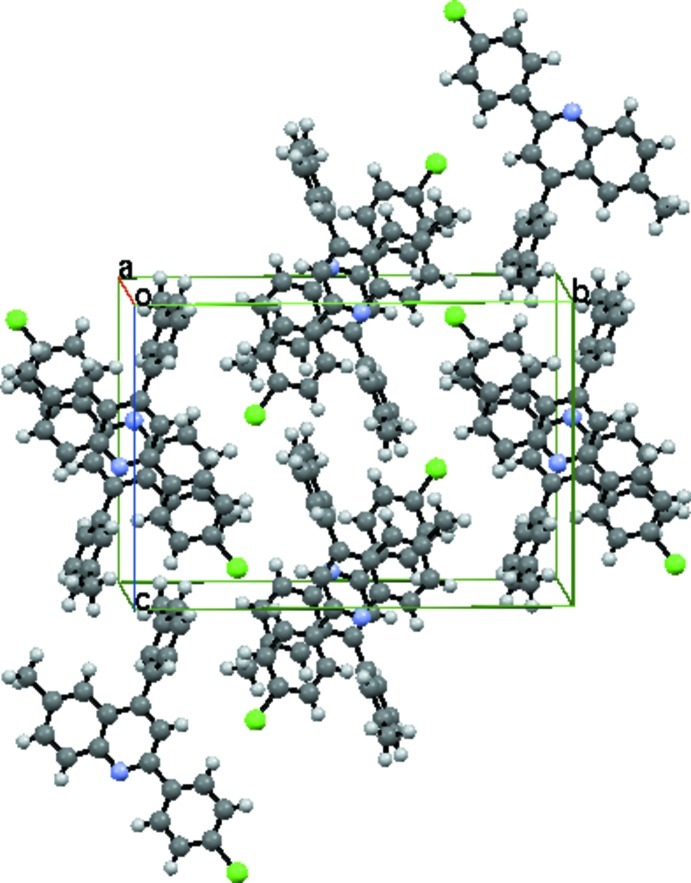
A packing diagram of the title compound, viewed along the crystallographic a axis.

## supplementary crystallographic information

### Comment

Quinolines and their derivatives occur in numerous natural products (Michael, 1997) and may exhibit interesting physiochemical activities, finding applications as pharmaceuticals and agrochemicals as well as being general synthetic platforms (Balasubramanian *et al.*, 1996).

In the title molecule, C_23_ H_18_ Cl N, (Fig. 1), dihedral angles between the quinoline moiety and the chlorobenzene and methylbenzene rings are 2.57 (9) and 56.06 (9)°, respectively, with the conformation of the chlorobenzene ring influenced by the presence of an intramolecular C5—H···N1 interaction [2.764 (3) Å]. The overall geometry of the title compound is similar to 4-(4-chlorophenyl)-8-methyl-2-oxo-1,2,5,6,7,8-hexahydroquinoline -3-carbonitrile (Asiri *et al.*, 2011).

The crystal structure (Fig. 2) is stabilized by aromatic ring *π*··· *π* interactions with the ring centroids defined as follows: *Cg*(1), N1/C7/C8/C9/C10/C15; *Cg*(2), C1/C2/C3/C4/C5/C6 and *Cg*(3), C10/C11/C12/C13/C14/C15. The distance between *Cg*(1) and *Cg*(1) is 3.7427 (18) Å [*-x*+3, *-y*+2, *-z*+1], *Cg*(1) and *Cg*(2) is 3.7679 (19) Å [*-x*+2,*-y*+2,1 -z], *Cg*(1) and *Cg*(3) is 3.7635 (18) Å [*-x*+3, *-y*+2, *-z*+1], *Cg*(2) and *Cg*(3) is 3.733 (2) Å [*-x*+2, *-y*+2, *-z*+1].

### Experimental

The enaminone [3-(4-chlorophenyl)-1-m-tolyl-3-(p-tolyamino)prop-2-en-1-one] (5 mmol) was taken in polyphosphoric acid (5 mL) and heated at 140 °C for 5 h. After completion of the reaction (monitored by TLC), the reaction mixture was diluted with water (50 mL). The aqueous layer was extracted with ethyl acetate (3 *x* 20 mL), the combined ethyl acetate layer was washed with 0.1 N NaOH (2 *x* 25 mL), followed by brine solution (25 mL). The organic layer was then dried over anhydrous sodium sulfate and concentrated under reduced pressure to afford the crude product which was purified by column chromatography over silica gel (60–120 mesh) using a hexane:ethyl acetate mixture (9.5:0.5) as eluent. The pure title compound was crystallized in an ethyl acetate–hexane mixture to obtain pale yellow single crystals. ^1^H NMR (CDCl_3_, 300 MHz): 8.49 (d, 2H, J=8.4 Hz), 7.92 (d, 1H, J=7.2 Hz), 7.79 (s, 1H), 7.55–7.66 (m, 5H), 7.19 (d, 1H), 2.34 (s, 3H), 2.35 (s, 3H). Mass: Calc. 343.2 found 344.2 (*M*^+^+1): m.p. 98–100 °C (uncorrected).

### Refinement

All hydrogen atoms were located geometrically with C—H = 0.93–0.97) Å and allowed to ride on their parent atoms with *U*_iso_(H) = 1.2*U*_eq_(aromatic C) or 1.5*U*_eq_(methyl C).

### Crystal data

**Table:**

C_23_H_18_ClN	*F*(000) = 720
*M**_r_* = 343.83	*D*_x_ = 1.281 Mg m^−^^3^
Monoclinic, *P*2_1_/*c*	Melting point = 371–373 K
Hall symbol: -P 2ybc	Mo *K*α radiation, λ = 0.71073 Å
*a* = 7.982 (3) Å	Cell parameters from 3392 reflections
*b* = 17.921 (6) Å	θ = 2.0–25.7°
*c* = 12.478 (4) Å	µ = 0.22 mm^−^^1^
β = 92.581 (6)°	*T* = 293 K
*V* = 1783.1 (11) Å^3^	Block, yellow
*Z* = 4	0.23 × 0.22 × 0.22 mm

### Data collection

**Table:**

Oxford Diffraction Xcalibur CCD diffractometer	2508 reflections with *I* > 2σ(*I*)
Radiation source: fine-focus sealed tube	*R*_int_ = 0.044
Graphite monochromator	θ_max_ = 25.7°, θ_min_ = 2.0°
Detector resolution: 16.0839 pixels mm^-1^	*h* = −9→9
ω scans	*k* = −21→21
16998 measured reflections	*l* = −15→15
3392 independent reflections	

### Refinement

**Table:**

Refinement on *F*^2^	Primary atom site location: structure-invariant direct methods
Least-squares matrix: full	Secondary atom site location: difference Fourier map
*R*[*F*^2^ > 2σ(*F*^2^)] = 0.057	Hydrogen site location: inferred from neighbouring sites
*wR*(*F*^2^) = 0.168	H-atom parameters constrained
*S* = 1.04	*w* = 1/[σ^2^(*F*_o_^2^) + (0.0922*P*)^2^ + 0.5751*P*] *P* = (*F*_o_^2^ + 2*F*_c_^2^)/3
3392 reflections	(Δ/σ)_max_ = 0.001
228 parameters	Δρ_max_ = 0.49 e Å^−^^3^
0 restraints	Δρ_min_ = −0.45 e Å^−^^3^

### Special details

**Table:**

Geometry. Bond distances, angles *etc*. have been calculated using the rounded fractional coordinates. All su's are estimated from the variances of the (full) variance-covariance matrix. The cell e.s.d.'s are taken into account in the estimation of distances, angles and torsion angles
Refinement. Refinement on *F*^2^ for ALL reflections except those flagged by the user for potential systematic errors. Weighted *R*-factors *wR* and all goodnesses of fit *S* are based on *F*^2^, conventional *R*-factors *R* are based on *F*, with *F* set to zero for negative *F*^2^. The observed criterion of *F*^2^ > σ(*F*^2^) is used only for calculating -*R*-factor-obs *etc*. and is not relevant to the choice of reflections for refinement. *R*-factors based on *F*^2^ are statistically about twice as large as those based on *F*, and *R*-factors based on ALL data will be even larger.

### Fractional atomic coordinates and isotropic or equivalent isotropic displacement parameters (Å^2^)

**Table:**

	*x*	*y*	*z*	*U*_iso_*/*U*_eq_	
Cl1	0.89231 (10)	1.22893 (5)	0.85463 (6)	0.0816 (3)	
N1	1.2557 (2)	0.97450 (10)	0.55301 (13)	0.0406 (6)	
C1	0.9786 (3)	1.17421 (15)	0.7563 (2)	0.0542 (8)	
C2	0.9969 (4)	1.20263 (15)	0.6569 (2)	0.0640 (9)	
C3	1.0682 (4)	1.15951 (14)	0.5796 (2)	0.0607 (9)	
C4	1.1224 (3)	1.08789 (12)	0.60147 (17)	0.0404 (7)	
C5	1.0989 (3)	1.06052 (14)	0.70260 (18)	0.0510 (8)	
C6	1.0278 (3)	1.10238 (15)	0.7802 (2)	0.0590 (9)	
C7	1.2017 (2)	1.04084 (11)	0.52008 (17)	0.0387 (6)	
C8	1.2226 (3)	1.06583 (11)	0.41476 (17)	0.0411 (6)	
C9	1.2984 (3)	1.02293 (11)	0.34173 (16)	0.0380 (6)	
C10	1.3536 (2)	0.95017 (11)	0.37377 (16)	0.0369 (6)	
C11	1.4245 (3)	0.89791 (11)	0.30488 (18)	0.0413 (7)	
C12	1.4739 (3)	0.82860 (11)	0.33979 (19)	0.0451 (7)	
C13	1.4533 (3)	0.81027 (12)	0.44755 (19)	0.0512 (8)	
C14	1.3836 (3)	0.85844 (12)	0.51636 (19)	0.0486 (7)	
C15	1.3289 (2)	0.92982 (11)	0.48076 (16)	0.0379 (6)	
C16	1.3247 (3)	1.05256 (11)	0.23256 (16)	0.0417 (7)	
C17	1.1911 (3)	1.08107 (13)	0.17090 (18)	0.0523 (8)	
C18	1.2162 (4)	1.10960 (14)	0.0705 (2)	0.0630 (10)	
C19	1.3729 (4)	1.11009 (13)	0.03096 (19)	0.0639 (9)	
C20	1.5098 (4)	1.08379 (13)	0.0910 (2)	0.0555 (8)	
C21	1.4827 (3)	1.05492 (11)	0.19201 (18)	0.0467 (7)	
C22	1.6837 (4)	1.08762 (18)	0.0504 (3)	0.0827 (11)	
C23	1.5477 (4)	0.77324 (13)	0.2648 (2)	0.0612 (9)	
H2	0.96150	1.25100	0.64090	0.0770*	
H3	1.07990	1.17910	0.51130	0.0730*	
H5	1.13240	1.01200	0.71880	0.0610*	
H6	1.01310	1.08260	0.84800	0.0710*	
H8	1.18350	1.11290	0.39470	0.0490*	
H11	1.43820	0.91080	0.23360	0.0500*	
H13	1.48850	0.76370	0.47260	0.0610*	
H14	1.37150	0.84460	0.58740	0.0580*	
H17	1.08400	1.08090	0.19740	0.0630*	
H18	1.12600	1.12860	0.02940	0.0750*	
H19	1.38760	1.12850	−0.03770	0.0770*	
H21	1.57340	1.03670	0.23350	0.0560*	
H22A	1.75030	1.04790	0.08160	0.1240*	
H22B	1.73340	1.13470	0.06980	0.1240*	
H22C	1.67850	1.08260	−0.02630	0.1240*	
H23A	1.47600	0.73030	0.25820	0.0920*	
H23B	1.65660	0.75820	0.29280	0.0920*	
H23C	1.55780	0.79580	0.19560	0.0920*	

### Atomic displacement parameters (Å^2^)

**Table:**

	*U*^11^	*U*^22^	*U*^33^	*U*^12^	*U*^13^	*U*^23^
Cl1	0.0855 (6)	0.0839 (6)	0.0764 (5)	0.0060 (4)	0.0149 (4)	−0.0363 (4)
N1	0.0447 (10)	0.0354 (9)	0.0415 (10)	−0.0025 (8)	0.0002 (8)	0.0039 (7)
C1	0.0449 (13)	0.0615 (16)	0.0562 (14)	−0.0035 (11)	0.0027 (11)	−0.0205 (12)
C2	0.0766 (18)	0.0471 (14)	0.0690 (17)	0.0126 (13)	0.0111 (14)	−0.0059 (13)
C3	0.0819 (19)	0.0481 (14)	0.0527 (14)	0.0151 (13)	0.0097 (13)	0.0048 (11)
C4	0.0347 (11)	0.0415 (12)	0.0448 (12)	−0.0022 (9)	−0.0009 (9)	−0.0023 (9)
C5	0.0576 (14)	0.0497 (13)	0.0457 (13)	0.0044 (11)	0.0039 (10)	0.0013 (10)
C6	0.0653 (16)	0.0642 (16)	0.0477 (13)	0.0006 (13)	0.0060 (12)	−0.0022 (11)
C7	0.0359 (11)	0.0364 (11)	0.0433 (11)	−0.0029 (8)	−0.0032 (9)	0.0012 (9)
C8	0.0463 (12)	0.0325 (10)	0.0441 (11)	0.0039 (9)	−0.0012 (9)	0.0032 (9)
C9	0.0396 (11)	0.0340 (10)	0.0401 (11)	−0.0022 (9)	−0.0026 (9)	0.0023 (8)
C10	0.0356 (11)	0.0307 (10)	0.0438 (11)	−0.0037 (8)	−0.0036 (8)	0.0026 (8)
C11	0.0454 (12)	0.0345 (11)	0.0440 (11)	−0.0031 (9)	0.0015 (9)	0.0000 (9)
C12	0.0448 (13)	0.0326 (11)	0.0576 (13)	−0.0005 (9)	−0.0018 (10)	−0.0033 (10)
C13	0.0620 (15)	0.0300 (11)	0.0612 (15)	0.0031 (10)	−0.0007 (12)	0.0081 (10)
C14	0.0601 (14)	0.0359 (11)	0.0496 (13)	−0.0009 (10)	−0.0005 (11)	0.0109 (9)
C15	0.0370 (11)	0.0322 (10)	0.0441 (11)	−0.0042 (8)	−0.0016 (9)	0.0030 (8)
C16	0.0567 (13)	0.0268 (10)	0.0415 (11)	0.0019 (9)	0.0021 (10)	0.0002 (8)
C17	0.0626 (16)	0.0441 (13)	0.0495 (13)	0.0051 (11)	−0.0037 (11)	0.0025 (10)
C18	0.089 (2)	0.0514 (15)	0.0472 (14)	0.0086 (13)	−0.0120 (14)	0.0055 (11)
C19	0.112 (2)	0.0409 (13)	0.0391 (12)	−0.0041 (14)	0.0070 (14)	0.0062 (10)
C20	0.0813 (18)	0.0334 (11)	0.0529 (14)	−0.0071 (11)	0.0155 (13)	−0.0022 (10)
C21	0.0614 (14)	0.0315 (11)	0.0476 (12)	0.0009 (10)	0.0061 (10)	0.0026 (9)
C22	0.102 (2)	0.0675 (18)	0.082 (2)	−0.0143 (17)	0.0404 (18)	0.0048 (16)
C23	0.0752 (18)	0.0396 (13)	0.0686 (17)	0.0096 (12)	0.0006 (14)	−0.0068 (11)

### Geometric parameters (Å, º)

**Table:**

Cl1—C1	1.737 (3)	C17—C18	1.376 (3)
N1—C7	1.324 (3)	C18—C19	1.365 (4)
N1—C15	1.358 (3)	C19—C20	1.380 (4)
C1—C2	1.355 (4)	C20—C21	1.388 (3)
C1—C6	1.375 (4)	C20—C22	1.501 (5)
C2—C3	1.379 (4)	C2—H2	0.9300
C3—C4	1.378 (3)	C3—H3	0.9300
C4—C5	1.375 (3)	C5—H5	0.9300
C4—C7	1.484 (3)	C6—H6	0.9300
C5—C6	1.368 (3)	C8—H8	0.9300
C7—C8	1.405 (3)	C11—H11	0.9300
C8—C9	1.355 (3)	C13—H13	0.9300
C9—C10	1.428 (3)	C14—H14	0.9300
C9—C16	1.486 (3)	C17—H17	0.9300
C10—C11	1.407 (3)	C18—H18	0.9300
C10—C15	1.406 (3)	C19—H19	0.9300
C11—C12	1.368 (3)	C21—H21	0.9300
C12—C13	1.401 (3)	C22—H22A	0.9600
C12—C23	1.502 (3)	C22—H22B	0.9600
C13—C14	1.355 (3)	C22—H22C	0.9600
C14—C15	1.417 (3)	C23—H23A	0.9600
C16—C17	1.384 (3)	C23—H23B	0.9600
C16—C21	1.381 (3)	C23—H23C	0.9600
			
C7—N1—C15	117.87 (17)	C21—C20—C22	120.5 (3)
Cl1—C1—C2	119.8 (2)	C16—C21—C20	121.8 (2)
Cl1—C1—C6	119.60 (19)	C1—C2—H2	120.00
C2—C1—C6	120.6 (2)	C3—C2—H2	120.00
C1—C2—C3	119.7 (2)	C2—C3—H3	119.00
C2—C3—C4	121.3 (2)	C4—C3—H3	119.00
C3—C4—C5	117.4 (2)	C4—C5—H5	119.00
C3—C4—C7	122.3 (2)	C6—C5—H5	119.00
C5—C4—C7	120.4 (2)	C1—C6—H6	121.00
C4—C5—C6	122.2 (2)	C5—C6—H6	121.00
C1—C6—C5	118.9 (2)	C7—C8—H8	119.00
N1—C7—C4	116.17 (18)	C9—C8—H8	119.00
N1—C7—C8	121.72 (18)	C10—C11—H11	119.00
C4—C7—C8	122.09 (18)	C12—C11—H11	119.00
C7—C8—C9	121.55 (19)	C12—C13—H13	119.00
C8—C9—C10	118.12 (18)	C14—C13—H13	119.00
C8—C9—C16	119.97 (18)	C13—C14—H14	120.00
C10—C9—C16	121.91 (18)	C15—C14—H14	120.00
C9—C10—C11	124.36 (19)	C16—C17—H17	120.00
C9—C10—C15	116.61 (17)	C18—C17—H17	120.00
C11—C10—C15	119.00 (18)	C17—C18—H18	120.00
C10—C11—C12	121.9 (2)	C19—C18—H18	120.00
C11—C12—C13	118.3 (2)	C18—C19—H19	119.00
C11—C12—C23	121.1 (2)	C20—C19—H19	119.00
C13—C12—C23	120.61 (19)	C16—C21—H21	119.00
C12—C13—C14	121.9 (2)	C20—C21—H21	119.00
C13—C14—C15	120.4 (2)	C20—C22—H22A	109.00
N1—C15—C10	124.08 (18)	C20—C22—H22B	109.00
N1—C15—C14	117.40 (18)	C20—C22—H22C	109.00
C10—C15—C14	118.52 (18)	H22A—C22—H22B	110.00
C9—C16—C17	120.3 (2)	H22A—C22—H22C	109.00
C9—C16—C21	121.1 (2)	H22B—C22—H22C	109.00
C17—C16—C21	118.6 (2)	C12—C23—H23A	109.00
C16—C17—C18	120.2 (2)	C12—C23—H23B	109.00
C17—C18—C19	120.3 (3)	C12—C23—H23C	109.00
C18—C19—C20	121.2 (2)	H23A—C23—H23B	109.00
C19—C20—C21	117.9 (3)	H23A—C23—H23C	110.00
C19—C20—C22	121.5 (2)	H23B—C23—H23C	110.00
			
C15—N1—C7—C8	−1.7 (3)	C8—C9—C10—C11	175.9 (2)
C7—N1—C15—C14	−178.93 (18)	C10—C9—C16—C21	−54.9 (3)
C7—N1—C15—C10	1.2 (3)	C10—C9—C16—C17	127.5 (2)
C15—N1—C7—C4	179.95 (17)	C15—C10—C11—C12	−1.7 (3)
Cl1—C1—C6—C5	−178.59 (19)	C9—C10—C15—N1	0.6 (3)
Cl1—C1—C2—C3	178.8 (2)	C11—C10—C15—N1	−177.30 (18)
C6—C1—C2—C3	−1.1 (4)	C11—C10—C15—C14	2.8 (3)
C2—C1—C6—C5	1.4 (4)	C9—C10—C15—C14	−179.27 (19)
C1—C2—C3—C4	−0.4 (5)	C9—C10—C11—C12	−179.4 (2)
C2—C3—C4—C7	−179.1 (2)	C10—C11—C12—C23	179.4 (2)
C2—C3—C4—C5	1.5 (4)	C10—C11—C12—C13	−0.4 (3)
C3—C4—C7—N1	176.7 (2)	C23—C12—C13—C14	−178.5 (2)
C5—C4—C7—C8	177.7 (2)	C11—C12—C13—C14	1.3 (4)
C5—C4—C7—N1	−3.9 (3)	C12—C13—C14—C15	−0.1 (4)
C3—C4—C5—C6	−1.3 (4)	C13—C14—C15—C10	−2.0 (3)
C7—C4—C5—C6	179.3 (2)	C13—C14—C15—N1	178.1 (2)
C3—C4—C7—C8	−1.6 (3)	C9—C16—C17—C18	178.9 (2)
C4—C5—C6—C1	−0.1 (4)	C21—C16—C17—C18	1.3 (3)
N1—C7—C8—C9	0.3 (3)	C9—C16—C21—C20	−178.7 (2)
C4—C7—C8—C9	178.6 (2)	C17—C16—C21—C20	−1.1 (3)
C7—C8—C9—C10	1.5 (3)	C16—C17—C18—C19	−0.1 (4)
C7—C8—C9—C16	−177.4 (2)	C17—C18—C19—C20	−1.5 (4)
C8—C9—C10—C15	−1.9 (3)	C18—C19—C20—C21	1.7 (4)
C16—C9—C10—C11	−5.3 (3)	C18—C19—C20—C22	−176.9 (2)
C8—C9—C16—C17	−53.6 (3)	C19—C20—C21—C16	−0.4 (3)
C8—C9—C16—C21	123.9 (2)	C22—C20—C21—C16	178.2 (2)
C16—C9—C10—C15	176.97 (19)		

### Hydrogen-bond geometry (Å, º)

**Table:**

*D*—H···*A*	*D*—H	H···*A*	*D*···*A*	*D*—H···*A*
C5—H5···N1	0.93	2.43	2.764 (3)	101
